# CX3CL1/CX3CR1 and CCL2/CCR2 Chemokine/Chemokine Receptor Complex in Patients with AMD

**DOI:** 10.1371/journal.pone.0112473

**Published:** 2014-12-15

**Authors:** Mads Krüger Falk, Amardeep Singh, Carsten Faber, Mogens Holst Nissen, Thomas Hviid, Torben Lykke Sørensen

**Affiliations:** 1 Clinical Eye Research Unit, Department of Ophthalmology, Copenhagen University Hospital Roskilde, Roskilde, Denmark and Faculty of Health Sciences, University of Copenhagen, Copenhagen, Denmark; 2 Department of Microbiology, Immunology & International Health, Faculty of Health Sciences, University of Copenhagen, Copenhagen, Denmark; 3 Centre for Immune Regulation and Reproductive Immunology (CIRRI), Department of Clinical Biochemistry, Copenhagen University Hospital Roskilde, Roskilde, Denmark and Faculty of Health Sciences, University of Copenhagen, Copenhagen, Denmark; 4 Department of Ophthalmology, Glostrup Hospital, Glostrup, Denmark; Institut de la Vision, France

## Abstract

**Purpose:**

The chemokine receptors CX3CR1 and CCR2 have been implicated in the development of age-related macular degeneration (AMD). The evidence is mainly derived from experimental cell studies and murine models of AMD. The purpose of this study was to investigate the association between expression of CX3CR1 and CCR2 on different leukocyte subsets and AMD. Furthermore we measured the plasma levels of ligands CX3CL1 and CCL2.

**Methods:**

Patients attending our department were asked to participate in the study. The diagnosis of AMD was based on clinical examination and multimodal imaging techniques. Chemokine plasma level and chemokine receptor expression were measured by flow-cytometry.

**Results:**

A total of 150 participants were included. We found a significantly lower expression of CX3CR1 on CD8^+^ T cells in the neovascular AMD group compared to the control group (p = 0.04). We found a significant positive correlation between CCR2 and CX3CR1 expression on CD8^+^ cells (r = 0.727, p = 0.0001). We found no difference in plasma levels of CX3CL1 and CCL2 among the groups.

**Conclusions:**

Our results show a down regulation of CX3CR1 on CD8^+^ cells; this correlated to a low expression of CCR2 on CD8^+^ cells. Further studies are needed to elucidate the possible role of this cell type in AMD development.

## Introduction

Age-related macular degeneration (AMD) is a disease with a complex etiology. Inflammation is thought to be a major factor in development and progression of AMD. [1] Single nucleotide polymorphisms (SNP) in the complement factor H (CFH) gene are strongly related to development of AMD. [2] Furthermore, several chemokines have been suggested to be involved in the pathogenesis of AMD. [1], [3] Chemokines are families of small cytokines that share a common cysteine motif at the N-terminal end of the protein. Chemokines exert their function through chemokine receptors that are expressed on a great variety of cells throughout the body. [4] Traditionally chemokines were known for their chemotactic function, guiding lymphocytes to sites of inflammation. However, in recent years it has become clear that chemokines have a variety of other functions. [5] In AMD, an increasing body of evidence suggests that chemokines play a significant role in the development of AMD. [1] Chemokine receptors such as CCR3 and CXCR3 have been associated with development of neovascular AMD while others such as CCR2 and CX3CR1 have been associated with drusen formation and development of early AMD.[6]–[9] Most of the evidence derives from studies carried out on murine models of AMD or experimental cell studies. However, in recent years, studies on AMD patients investigating intraocular expression or peripheral expression of chemokines and receptors have also been presented.[10]–[12].

CCL2 and CX3CL1 signaling through CCR2 and CX3CR1 have shown to be key factors in recruitment of macrophages to tissue lesions or sites of inflammation. [13] There is evidence that CCR2 and CX3CR1 have an important role in trafficking microglia cells to and from the subretinal space. [7], [14], [15] Combadiére et* al.* showed that the lesions found in *CX3CR1* knock-out mice consisted of lipid bloated macrophages that accumulate subretinally probably due to deficiencies in the migratory properties. [7].

There is increasing evidence of CCR2 and CX3CR1 and their ligands, CCL2 and CX3CL1, being involved in the development and progression of AMD. Since most of the studies made in this area are carried out on mouse models of AMD and in experimental cell studies, we examined the peripheral expression of CCR2 and CX3CR1 on different lymphocyte subsets and measured the plasma levels of CCL2 and CX3CL1 in patients with AMD.

## Materials and Methods

### Participants

During a period of 20 months, patients with AMD attending our department were asked to participate in this case-control study. Individuals attending our department for other reasons were asked to participate as control subjects. AMD was diagnosed according to the Age-Related Eye Disease Study (AREDs) criteria. Participants were excluded from the study if they were diagnosed with malignant or autoimmune disease including type 1 diabetes. All participants in active treatment with immunosuppressive agents were excluded. To avoid interference from other acute-phase responses due to undiagnosed cancers and acute infections serum C-reactive protein (CRP) was measured in all participants. All participants having a serum level of CRP>10 mg/L were excluded.

The participants included were divided according to their AMD stage in the following groups: 1) Healthy controls; 2) Early AMD defined by the presence of drusen and 3) Neovascular AMD. None of the participants had signs of polypoidal vasculopathy, retinal angiomatous proliferation, or chorioretinal anastomosis. None of the participants had reticular drusen. All patients diagnosed with neovascular AMD were treatment naïve to Bevacizumab (Avastin, Roche, Basel, Switzerland), Aflibercept (Eylea, Bayer, Leverkusen, Germany) and had not received injection with Ranibizumab (Lucentis, Genentech, San Francisco, USA) for the last five weeks prior to inclusion.

All participants underwent a structured interview with focus on current and previously medical conditions and current medication, smoking habits and alcohol consumption. Patients were defined as current smokers, former smokers (more than 100 cigarettes during their lifetime) or never smokers. Alcohol consumption was graded according to the Danish National Board of Health’s recommendations (maximum 7 units and 14 units per week for women and men, respectively). Height and weight were measured at the first visit to calculate the body mass index (BMI).

Part of the study population described in this study has been described in previous studies.[10], [16]–[19].

Verbal and written informed consent was obtained from all participants prior to inclusion. The study has been approved by the Regional Committee of Ethics in Research of the Region of Zealand (SJ-142) and was performed in adherence to the Declaration of Helsinki.

### Ophthalmic examination

All participants underwent a thorough ophthalmologic examination including slitlamp biomicroscopy and fundoscopy, colour fundus photography (Carl Zeiss, Jena, Germany), Spectral-Domain-Optical Coherence Tomography (SD-OCT) and fundus autofluorescence imaging (Spectralis HRA-OCT, Heidelberg Engineering, Heidelberg, Germany). Fluorescein and indocyanine green (FA/ICG) angiography was performed on patients suspicious of neovascular AMD in order to obtain correct diagnosis. Visual acuity (VA) was measured using the Early Treatment Diabetic Retinopathy Study (ETDRS) chart. [20].

### Leukocyte preparation and flow cytometry

**Figure 1:**
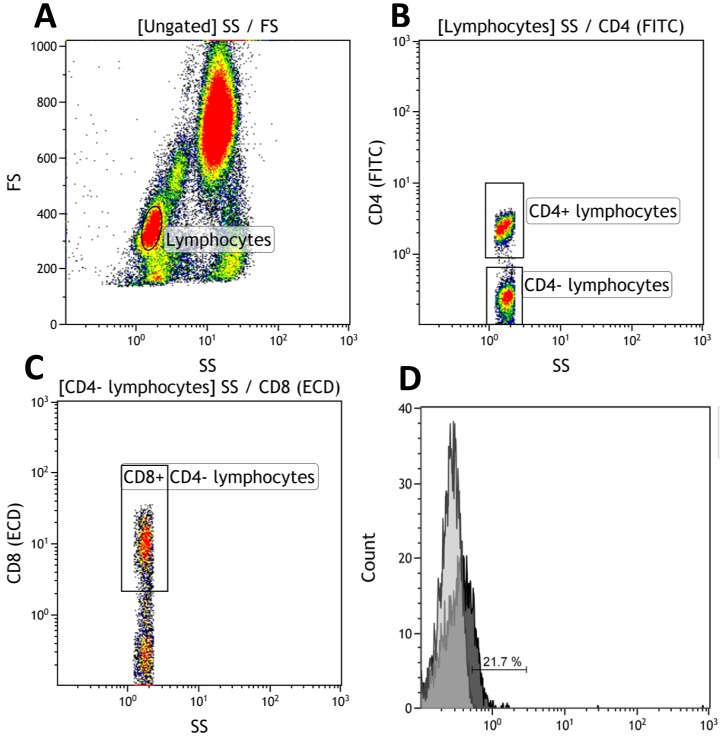
Gating strategy and quantification of CD8^+^ T cells and their expression of CX3CR1. A, Lymphocytes were identified based on forward scatter and side scatter. B, the lymphocytes were further gated based on their CD4 expression. C, The subset of CD4^-^ T cells that are CD8 negative is found. D, The fraction of CX3CR1^+^ cells within the CD4^−^ CD8^+^ T cells was calculated. Histogram showing the percentage of CD8^+^ T cells positive for CX3CR1 (dark grey) and the negative isotype control set at 1% (light grey).

Venous blood samples were obtained from all participants on the day of inclusion and the blood samples were prepared for flow cytometry within 4 hours of phlebotomy. In patients undergoing retinal angiography, phlebotomy was performed prior to angiography in order to avoid possible interference. [21] The blood samples were prepared for flow cytometry by the following steps. Red blood cell lysis was performed by adding 10% red blood cell lysis buffer (Biolegend, San Diego, CA, USA) to the whole blood and stored for 10 minutes in the dark at room temperature. After red blood cell lysis the sample was washed three times in isotonic buffer (IsoFlow Sheath Fluid Beckman Coulter, Brea, CA, USA) and centrifuged for five minutes at 500G. After washing, the cells were incubated at room temperature in the dark for 25 minutes with mixtures of the following monoclonal anti-human antibodies: CD4 (IgG1, clone: 13B8.2, Beckman Coulter, Brea, CA, USA), CD8 (IgG1, clone: SFCI21Thy2D3, Beckman Coulter, Brea, CA, USA), CD25 (IgG2a, clone: B1.49.9, Beckman Coulter, Brea, CA, USA), CD69 (IgG2b, clone: TP1.55.3, Beckman Coulter, Brea, CA, USA), CD14 (IgG2a, clone: RMO52, Beckman Coulter, Brea, CA, USA), CX3CR1 (IgG1, clone:528728, R&D Systems, Inc, Minneapolis, MN, USA), CCR2 (IgG2b, clone:48607, R&D Systems, Inc, Minneapolis, MN, USA). Corresponding negative isotype controls were used and set at 1%. Flow cytometry was performed on a Beckman Coulter FC 500 (Beckman Coulter, Brea, CA, USA) flow cytometer. An example of how gating was performed can be seen in Fig. 1.

### Quantification of CX3CL1 and CCR2

Heparinized blood samples were centrifuged for 15 minutes at 1000 G, and plasma was isolated and stored at −80°C until analysis were performed. CX3CL1 and CCL2 were measured on an LSR II flow cytometer using a Cytometric Bead Array (BD Biosciences, Franklin Lakes, NJ, USA) following the manufacture’s recommendations.

### Clinical response

To assess the clinical relevance of peripheral expression of CCR2 and CX3CR1 and CX3CL1 and CCL2 we looked at treatment response after the initial three anti-VEGF injections with Ranibizumab. It has been suggested, that VA at this point of treatment is a useful predictor (among others) of treatment response. [22] Patients in active treatment with Ranibizumab were divided in three groups according to the change in visual acuity (VA) between the first visit and the follow-up visit after the initial three intravitreal injections. The three groups were: 1) Gain of more than 10 ETDRS letters 2) VA change (gain or loss) of maximum 10 ETDRS letters. 3) loss of more than 10 ETDRS letters and.

### Statistical analysis

The statistical software SPSS version 20 for Windows (IBM, Chicago, IL USA) was used for statistical analysis. Kruskal-Wallis test was used for one-way analysis of variance and Mann-Whitney U test was used to compare 2 groups following analysis of variance for not normally distributed continuous variables (CX3CR1, CCR2, CX3CL1, CCL2, age and BMI) and data are given as medians and interquartile ranges (IQR). Categorical variables were analyzed using Pearson Chi-square test (gender, alcohol-intake, smoking habits). Spearman correlation coefficient was used to examine the relationship between expression level of CCR2 and CX3CR1 on CD8^+^ T cells. The study population was divided in tertiles based on the expression of CX3CR1 and CCR2 on CD8^+^ cells. Odds Ratios (OR) for the prevalence of AMD were calculated by comparing the highest and lowest tertile for each receptor. A p-level of less than 0.05 was considered significant. A power calculation showed that a sample size of 24 in each group was needed to reach a power level of 80% and a statistical significance level of 0.05.

## Results

### Demographic data and clinical characteristics

A total of 150 participants were included in the study. Of these, 90 patients had neovascular AMD and 30 patients had early AMD. Thirty age-matched control persons were included. The clinical and demographic data can be seen in Table 1. No significant difference in age, gender, BMI, smoking habits or alcohol consumption was found between the groups.

**Table 1:** Demographic data on participants in the study.

	Age matched Controls (n = 30)	Early AMD (n = 30)	nAMD (n = 90)	P-value	Test
Male %	52	46	44	0.682	Pearson Chi
Female %	48	54	56		
Median Age (IQR)	77.0 (73.0;80.0)	77.5 (72.0;82.0)	75.0 (71.0;81.0)	0.474	Kruskal-Wallis
Current smokers (%yes)	10	17	19	0.817	Pearson Chi
Former smokers (%yes)	45	41	45		
Never smokers (%yes)	45	42	36		
Alcohol above recommended (%yes)	27	6	13	0.258	Pearson Chi
BMI median (IQR)	26.0 (23.0;28.0)	25.0(22.0;27.0)	25.0(22.5;28.0)	0.734	Kruskal-Wallis

Kruskal-Wallis test, a one-way analysis of variance was used to calculate differences among groups for not normally distributed continuous and data are given as medians and interquartile ranges (IQR). Categorical variables were analyzed using Pearson Chi-square test. P-values are given.

AMD, Age-related Macular Degeneration; nAMD, neovascular AMD; IQR, Inter Quartile Range; BMI, Body Mass Index.

### Expression of CX3CR1 and CCR2

**Figure 2:**
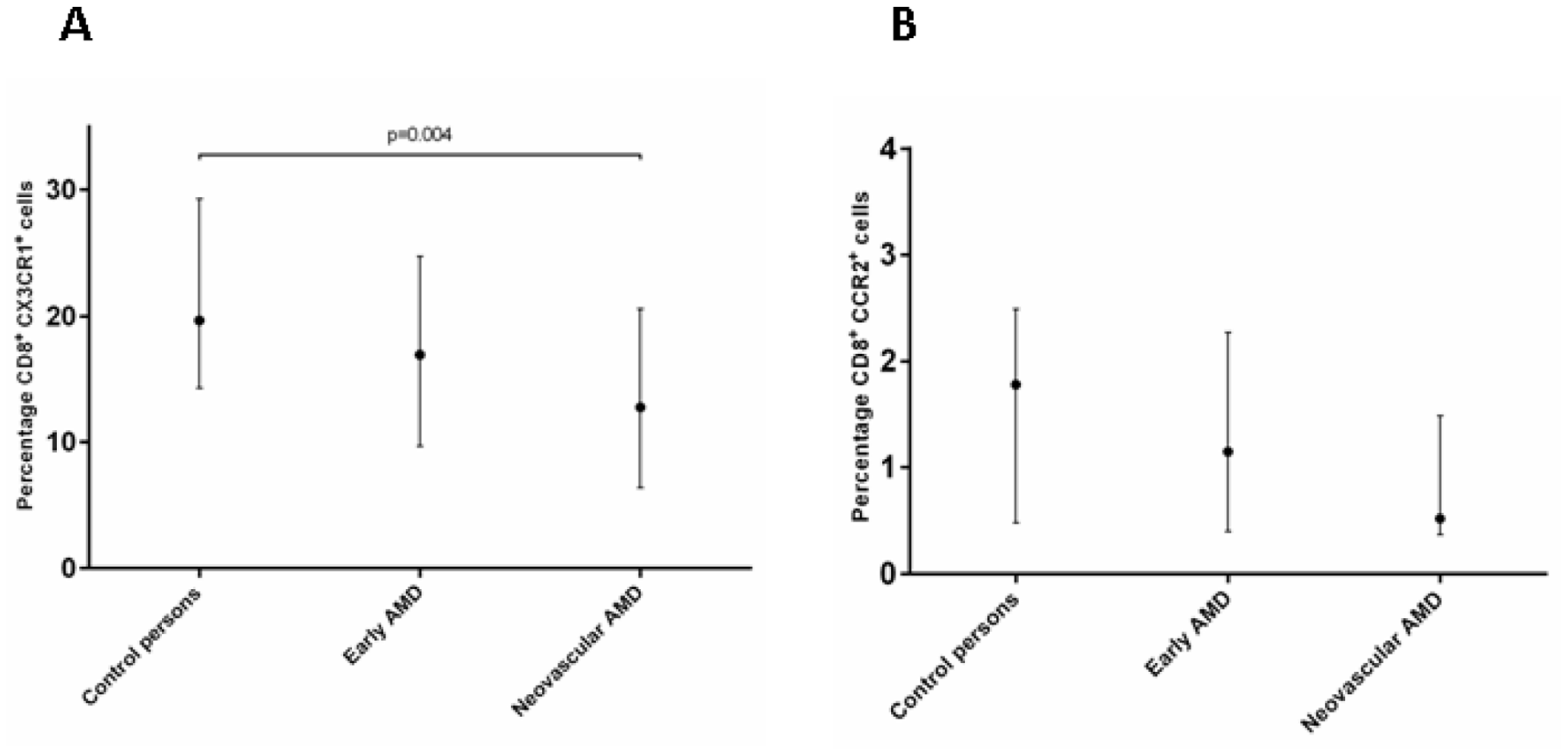
Expression of CX3CR1 and CCR2 on CD8+ cells. A: Percentage of CD8^+^ cells expressing CX3CR1 in the 150 patients included in the study. The dots represent medians and the lines denote inter quartile ranges (IQR). P-values, Mann-Whitney U Test. B: Percentage of CD8^+^ cells expressing CCR2 in the 150 patients included in the study. The dots represent medians and the lines denote inter quartile ranges (IQR).

**Figure 3:**
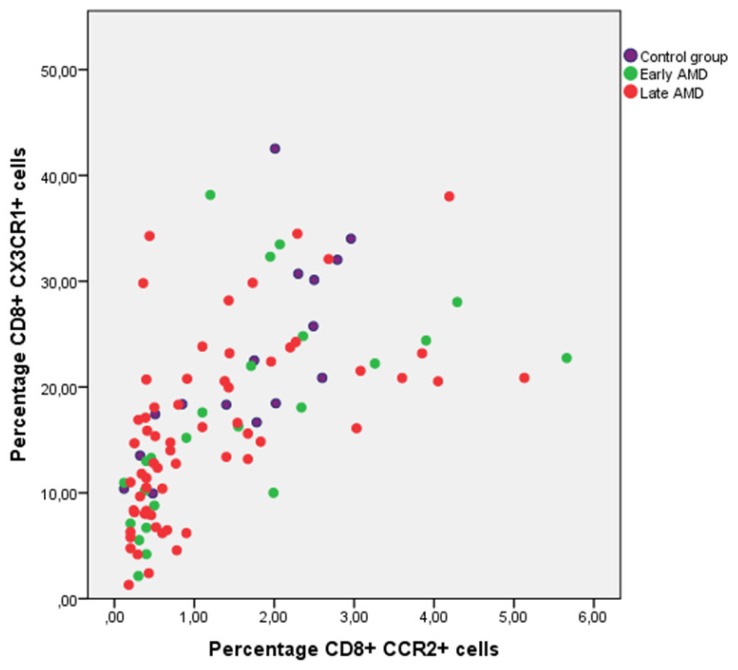
Correlation between expression of CCR2 and CX3CR1 on CD8^+^ cells. The plot shows a positive correlation between CCR2+ and CX3CR1 expression on CD8^+^ cells. The majority of participants with combined low frequency of CCR2 and CX3CR1 on CD8 cells were diagnosed with AMD. Combined CCR2 and CX3CR1 receptor expression data was available in 102 patients.

CX3CR1 is expressed on a subset of T cells and is important for the cells ability to migrate to sites of inflammation. [23], [24] Since both have been suggested to play a role in the pathogenesis of AMD we wanted to see whether we could find an alteration in expression of these chemokinereceptors on T cells. The expression of CX3CR1 and CCR2 was measured on CD4^+^ and CD8^+^ T cells. The data is shown in Table 2 and Fig. 2. We found a significantly lower frequency of CD8^+^ T cells expressing CX3CR1 in the neovascular AMD group compared to the control group (p = 0.04, Mann-Whitney U). Similarly, we found a tendency to lower frequency of CD8^+^ T cells expressing CCR2 in the group with neovascular AMD compared to the control (p = 0.150, Mann-Whitney U). We found a significant positive correlation between percentage of CD8^+^ CCR2^+^ cells and CD8^+^ CX3CR1^+^ cells (r = 0.727, p = 0.0001). To see whether the AMD prevalence was influenced by expression of CX3CR1 and CCR2 on CD8^+^ cells, we divided the study population in tertiles according to frequency of CCR2 and CX3CR1 expression on CD8^+^ cells as previously described. We found that patients with CX3CR1 expression in the lowest tertile had a crude OR for AMD (early and late stage) prevalence of 5.3 (95% confidence interval (CI) 1.1–25.2). Fig. 3 shows a plot of CD8^+^CCR2^+^ frequency against CD8^+^CX3CR1^+^ frequency. Combined CCR2 and CX3CR1 receptor expression data was available in 104 patients. The plot shows that almost all of the study persons with combined low frequency of CCR2 and CX3CR1 on CD8 cells were diagnosed with AMD.

**Table 2:** Percentage of cells expressing CCR2 or CX3CR1 among the different groups of study participants.

	Age-matched controls (n = 30)	Early AMD (n = 30)	nAMD (n = 90)	P-value Kruskal- Wallis
**CD4^+^ CCR2^+^ Median percentage (IQR) [median number]**	2.2 (1.1;2.6) [111]	1.8 (1.2;4.0) [213]	2.2 (1.1;2.8) [159]	0.720
**CD4^+^ CX3CR1^+^ Median (IQR) [median number]**	33.2 (11.3;54.4) [2968]	43.1 (22.6;61.0) [3192]	45.8 (18.8;60.2) [3408]	0.408
**CD8^+^ CCR2^+^ Median percentage (IQR) [median number]**	1.8 (0.5;2.5) [20]	1.2 (0.4;2.3) [32]	0.52 (0.4;1.5) [22]	0.150
**CD8^+^ CX3CR1^+^ Median percentage (IQR) [median number]**	19.7 (14.3;29.3) [656]	16.9 (9.7;24.7) [539]	12.8 (6.4;20.6) [647]	0.006
**CD14^+^ CCR2^+^ Median percentage (IQR) [median number]**	84.8 (83.3;86.8) [3454]	85.6 (84.9;86.6) [4258]	83.8 (77.6;89.3) [2716]	0.373
**CD14^+^ CX3CR1^+^ Median percentage (IQR) [median number]**	15.2 (9.8;23.1) [530]	11.3 (5.0;34.9) [470]	12.9 (7.1;26.9) [499]	0.876
**CD14+ CCR2^high^ CX3CR1^low^ Median percentage (IQR)** **[median number]**	50.7 (28.8;89.7) [226]	65.1 (12.8;80.9) [86]	53.9 (28.1;77.4) [340]	0.612
**CD14+ CCR2^mid^ CX3CR1^high^ Median percentage (IQR)** **[median number]**	4.2 (1.8;7.6) [321]	3.7 (1.9;8.2) [65]	3.5 (1.4;8.0) [71]	0.840
**CD14+ CCR2^low^ CX3CR1^high^ Median percentage (IQR)** **[median number]**	4.9 (3.8;10.2) [370]	6.8 (3.9;18.5) [68]	6.4 (1.3;12.2) [42]	0.509

Data are given as median and interquartile range (IQR). Kruskal-Wallis test, a one-way analysis of variance was used to calculate differences among groups. P-values are given.

AMD, Age-related Macular Degeneration; nAMD, neovascular AMD; IQR, Inter Quartile Range.

Monocytes can be differentiated in different functional phenotypes according to their expression of CX3CR1 and CCR2. [25] We divided the monocytes in three groups: 1. Classical (phagocytotic) monocytes being CCR2^high^ CX3CR1^low^ 2. Intermediate (pro-inflammatory) monocytes being CCR2^mid^ CX3CR1^high^ and 3. Non-classical (patrolling) monocytes being CCR2^low^ CX3CR1^high^. The data is shown in Table 2. No difference in frequency of the different monocytes was found between the groups.

### Plasma level of CX3CL1 and CCL2

The plasma levels of CX3CL1 and CCL2 were measured in all participants in the study. CX3CL1 is a chemokine found in the blood as membrane bound to different cell types but also as a soluble non-bound protein. [26] Only the soluble fraction of CX3CL1 was measured. We did not find any difference in CCL2 (p = 0.660) or CX3CL1 (p = 0.566) among the different groups of participants included in the study (Table 3).

**Table 3:** Plasma level of CX3CL1 and CCL2 among the different groups of study participants.

	Age-matched controls (n = 30)	Early AMD (n = 30)	nAMD (n = 90)	P-value Kruskal- Wallis
**CX3CL1 Median (IQR)**	0.0 (0.0–96.8)	0.0 (0.0–220.5)	0.0 (0.0–88.1)	0.566
**CCL2 Median (IQR)**	117.0 (72.2–149.7)	93.1 (53.4–131.3)	117.1 (67.3–154.4)	0.660

Data are given as median and interquartile range (IQR). Kruskal-Wallis test, a one-way analysis of variance was used to calculate differences among groups. P-values are given.

AMD, Age-related Macular Degeneration; nAMD, neovascular AMD; IQR, Inter Quartile Range.

### Clinical response

**Figure 4:**
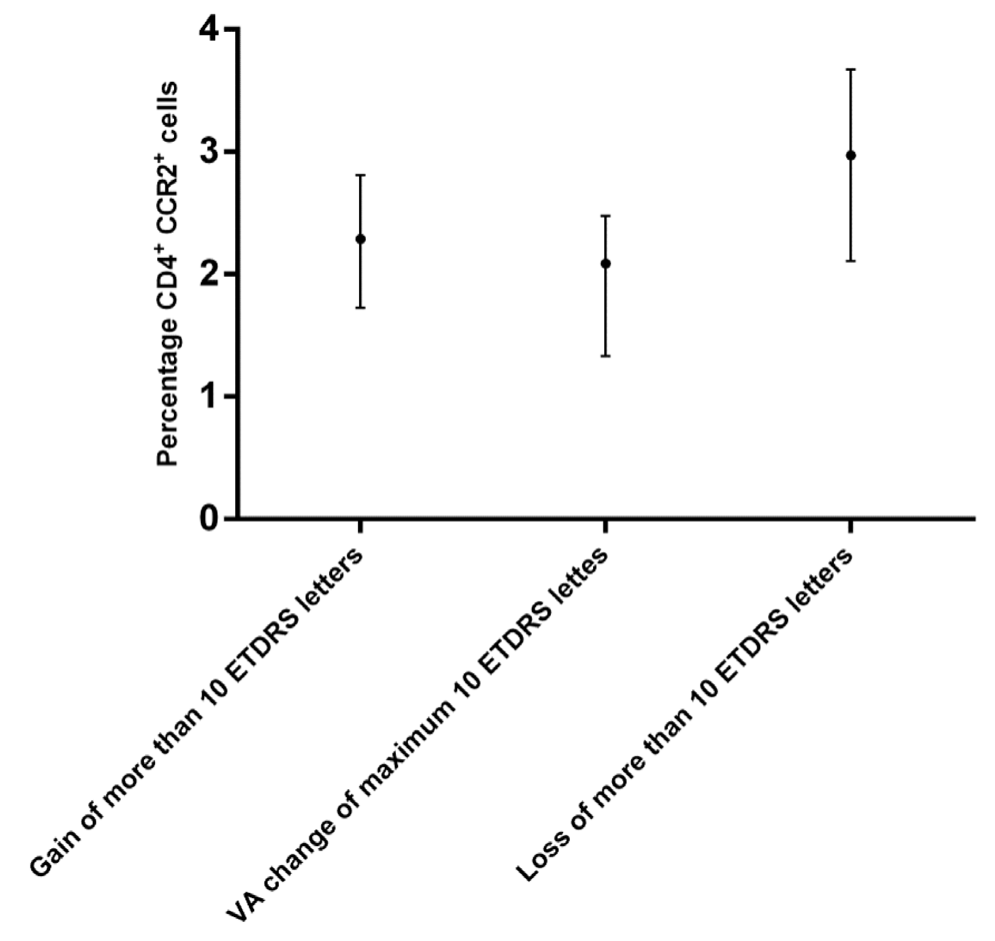
CCR2 expression and clinical response to anti-VEGF treatment. The patients with neovascular AMD were divided into groups according to change in visual acuity after the initial three anti-VEGF injections. The “Gain of more than 10 ETDRS letters” group consisted of 17 patients, the “VA change (gain or loss) of maximum 10 ETDRS letters” group consisted of 57 patient while the “loss of more than 10 ETDRS letters” group consisted of 12 patients. Percentage of CD4^+^ cells expressing CX3CR1 were calculated for each group. There was a tendency towards a higher expression of CCR2 on CD4+ cells in the group with a VA loss>10 ETDRS letters even though no significant change was found between the groups (p = 0.056, Kruskal-Wallis). The dots represent medians and the lines denote inter quartile ranges (IQR).

Patients in active treatment with Ranibizumab were divided in three groups according to their change in VA as described earlier. Data are presented in Table 4. We found a tendency towards a higher expression of CCR2 on CD4^+^ cells in the group with a VA loss of more than 10 ETDRS letters even though no significant change was found between the groups (p = 0.056, Kruskal-Wallis) (Fig. 4).

**Table 4:** Level of CCR2, CX3CR1, CCL2 and CX3CL1 according to clinical response to anti-VEGF treatment.

	Gain of more than 10 ETDRS letters (n = 17)	VA change of maximum 10 ETDRS letters (n = 57)	Loss of more than 10 ETDRS letters (n = 12)	P-value Kruskal-Wallis test
**CD4^+^ CCR2^+^ Median (IQR)**	2.26 (1.7;2.8)	2.1 (1.3;2.5)	2.9 (2.1;3.7)	0.056
**CD4^+^ CX3CR1^+^ Median (IQR)**	47.0 (30.8;54.7)	37.4 (11.6;57.7)	61.4 (19.0;77.1)	0.305
**CD8^+^ CCR2^+^ Median (IQR)**	0.4 (0.1;1.8)	0.4 (0.2;0.8)	0.7 (0.3;0.9)	0.616
**CD8^+^ CX3CR1^+^ Median (IQR)**	16.9 (9.1;24.1)	8.4 (3.4;17.8)	9.7 (4.3;19.3)	0.144
**CD14^+^ CCR2^+^ Median (IQR)**	84.9 (84.3;85.7)	85.6 (84.1;87.9)	83.3 (77.2;89.4)	0.553
**CD14^+^ CX3CR1^+^ Median (IQR)**	9.0 (2.6;36.4)	9.8 (2.4;23.9)	12.1 (1.1;27.1)	0.974
**CX3CL1 Median (IQR)**	0.0 (0.0;152.3)	0.0 (0.0;109.8)	27.8 (0.0;159.7)	0.780
**CCL2 Median (IQR)**	135.9 (62.4;182.1)	136.6 (63.2;172.5)	124.1 (45.3;159.7)	0.819
**CD14+ CCR2^high^ CX3CR1^low^ Median (IQR)**	42.0 (23.5;69.7)	53.9 (24.7;72.3)	65.3 (44.6;75.3)	0.513
**CD14+ CCR2^mid^ CX3CR1^high^ Median (IQR)**	4.9 (2.3;11.65)	3.4 (1.9;9.3)	2.7 (0.6;4.8)	0.389
**CD14+ CCR2^low^ CX3CR1^high^ Median (IQR)**	11.9 (3.11;20.9)	6.0 (2.8;12.4)	8.0 (2.9;10.2)	0.598

Patients with neovascular AMD in active treatment with anti-VEGF were divided in groups according to change in visual acuity after the three initial anti-VEGF injections. Plasma level of CCL2 and CX3CL1 and fraction (%) of cells expressing CCR2 or CX3CR1 were calculated for each group. Data are given as median and interquartile range (IQR). Kruskal-Wallis test, a one-way analysis of variance was used to calculate differences among groups. P-values are given.

VEGF, Vascular Endothelial Growth Factor; IQR, Inter Quartile Range; BMI, Body Mass Index; ETDRS, Early Treatment Diabetic Retinopathy Study.

## Discussion

Age-related macular degeneration is a disease with a complex etiology. Inflammation and dysregulation of inflammatory responses play an important role in development and progression of AMD. [1], [27] The involvement of different chemokines and chemokine receptors has been explored for more than a decade and yet the extent of their role in AMD is not fully understood. The chemokine receptors CCR2 and CX3CR1 have been implicated in the development of AMD. Especially murine models have shown that CCR2 and CX3CR1 and their ligands CCL2 and CX3CL1 are involved in drusen formation and RPE changes seen in the early stages of AMD. [6], [7], [28], [29].

It is believed that the main function of CCR2/CCL2 and CX3CR1/CX3CL1 is to recruit monocytes to sites of injury and inflammation. [13] Murine models of AMD have suggested that alterations in monocytes/macrophage function and recruitment in the retina are found in early stages of the disease. [6], [7].

Substantial evidence, points towards the CCR2/CCL2 receptor/ligand complex as an important factor in the development of AMD, even though contradictory results have come forward. Ambati et* al.* found that CCL2^−/−^ and CCR2^−/−^ knock-out mice develop many of the features found in early AMD. Thickening of Bruch’s membrane, swelling of the RPE cells and drusen-like lesions were found in the knock-out mice before the wild type mice developed these features. [6], [30] With increasing age the knock-out mice developed features of late stage AMD including CNV formation and atrophy of the RPE and photoreceptors. Whether this was due to the lack of CCR2/CCL2 or whether it was due to escalating uncontrolled inflammation in the retina caused by accumulating debris and lack of clearance is not known. In contrast to this Jonas et* al.* found that intraocular levels of CCL2 was increased in patients with neovascular AMD. [12] Recently Sennlaub and colleagues reported that CCL2 level and CCR2^+^ inflammatory infiltrating monocytes are increased in patients with geographic atrophy. [31] In a study using CCL2^−/−^ knock-out mice, Luhmann et* al.* found that the there was an age-related accumulation of bloated macrophages subretinally and that this process was accelerated in the CCL2^−/−^ knock-out mice model supporting previous findings by Xu et* al.* who found an age dependent subretinal accumulation of microglia, suggesting that there is a normal age-related increase in subretinal accumulating cells. [28], [32] Furthermore, Luhmann et* al.* did not find any spontaneous CNV formation in the ageing mice suggesting that the lack of CCL2 does not lead to CNV development. This is further supported by Tsutsumi et* al.* who found that laser-induced CNVs were smaller in CCR2^−/−^ knock-out mice compared with wild type mice. [33] Furthermore, Xie et* al.* showed that intravitreal injections with a CCR2 antagonist significantly could reduce the size of laser-induced CNV in mice. Moreover, the number of macrophages infiltrating the choroid decreased significantly in mice treated with the CCR2 antagonist, as well as a decrease in VEGF expression was seen in the treated mice. [29].

We measured the expression of CCR2 on different subsets of leukocytes but did not find any significant differences between the groups of participants. Nevertheless, there was a tendency towards a lower expression of CCR2 on CD8^+^ cells in the neovascular AMD group. CCL2 is not expressed at very high levels in the retina under normal conditions. [34] However, the expression level increases under certain conditions such as increasing age and oxidative stress, well known risk factors for AMD. [12], [35], [36] To see whether we could find a difference in plasma level of CCL2 we measured the plasma level in patients with AMD and control persons but did not find any difference. It is well known that expression and function of chemokines and receptors can diverge locally. Most of the above mentioned studies are performed on murine models of AMD and experimental cell studies. It cannot be expected that local changes in chemokine receptor expression is reflected systemically. Therefore, our results do not rule out CCR2 as a factor in developing AMD. Nevertheless, Grunin et* al.* reported a few years back that CCR2 and CCR1 was increased on a sub-type of monocytes in peripheral blood in patients with neovascular AMD. [11].

Like CCR2, the chemokine receptor CX3CR1 is important for the migration of leukocytes to sites of inflammation. Ng et* al.* proved the migratory properties of microglia in the retina of albino mice strains. [37] During light stimulus there were an accumulation of microglia and macrophages in the subretinal space. When the light stimulus was removed the microglia and macrophages were cleared. CX3CR1 is expressed by retinal microglia cells and studies have shown that CX3CR1 is important for the migratory properties of these cells in the retina. [7], [30] In CX3CR1^−/−^ knock-out mice microglia cells accumulate in the subretinal space with age after laser injury. The extended presence of microglia cells in the subretinal space results in excessive phagocytosis of outer segments, and the drusen-like lesions seen in CX3CR1 deficient mice have been found to be lipid bloated macrophages. [7] The accumulating microglia cells have a toxic effect on the RPE cells and photoreceptors and cause photoreceptor cells death. [14], [38] Since the microglia and macrophages produce pro-angiogenic factors and induce VEGF expression in RPE cells, it is possible that the accumulation of microglia and macrophages subretinally contributes to a more pro-angiogenic environment. [7], [39] We found that patients with neovascular AMD have a significantly lower percentage of CD8^+^ cells expressing CX3CR1 compared to healthy control persons. A lower expression of CX3CR1 is, as described previously, influencing the lymphocytic migratory properties. Down regulation of CX3CR1 expression could imply decreased ability of cells to migrate to sites of inflammation. This could be potentially beneficial since the recruitment of mononuclear cells to the subretinal space could lead to neurotoxic impact on the retina. At the same time lack of migratory properties could also trigger the inflammatory response in the retina because of the impaired trafficking of microglia cells within the retina. We measured the plasma level of CX3CL1 but did not find any difference between patients with AMD and control persons.

We found that patients with low percentages of CD8^+^ cells expressing CX3CR1 had a higher OR for developing late stage AMD compared to healthy controls. We found that the majority of patients having AMD and a low fraction of CX3CR1 expressing CD8^+^ cells, also had a low fraction of CD8^+^ cells expressing CCR2. A CCL2^−/−^ CX3CR1^−/−^ mouse model of AMD has been suggested, however contradictory findings have been published. [14], [40], [41].

We found a tendency towards higher expression of CCR2 on CD4^+^ cells in the group of patients with neovascular AMD who lost more than 10 ETDRS letters after the initial three anti-VEGF injections. It is possible that higher expression of CCR2 leads to a more pro-angiogenic environment, which in turn could result in a worse response to anti-VEGF treatment. It has been described that tissue expression of VEGF increases with increased CCR2 expression, and a synergistic effect on angiogenesis has been suggested. [42] However, Grunin et* al.* also looked at the clinical relevance of CCR2 and CCR1 expressed on a subset of monocytes and did not find any relationship between chemokine receptor expression and treatment response. [11].

The divergence in the reports about CCR2/CCL2 and CX3CR1/CX3CL1 involvement in development and progression of AMD is most likely a reflection of the complexity of the pathogenesis of AMD. The knock-out mice strains and murine models of AMD represents very regulated environments in contrast to the aging human body. In addition, chemokine receptors can be altered locally without this being reflected systemically. Therefore, our findings do not rule out CCR2 as a local factor in development of AMD, but indicates that this alteration in expression in the eye is not reflected systemically. Furthermore, it is unlikely that dysregulation of a single receptor or ligand can explain the development of AMD.

In conclusion, different murine models and experimental studies have revealed the involvement of CCR2 and CX3CR1 in the development and progression of AMD. Our findings also suggest a possible role for those chemokines in AMD pathogenesis, since we found a lower fraction of CX3CR1 expression on peripheral CD8^+^ cells in patients with neovascular AMD compared to control persons, and found that most of the patients with low expression of CX3CR1 and CCR2 on CD8^+^ T cells were diagnosed with AMD.
